# The necessity to choose causes the effects of reward on saccade preparation

**DOI:** 10.1038/s41598-017-17164-w

**Published:** 2017-12-05

**Authors:** Christian Wolf, Anna Heuer, Anna Schubö, Alexander C. Schütz

**Affiliations:** Experimental and Biological Psychology, Philipps-University Marburg, Marburg, Germany

## Abstract

When humans have to choose between different options, they can maximize their payoff by choosing the option that yields the highest reward. Information about reward is not only used to optimize decisions but also for movement preparation to minimize reaction times to rewarded targets. Here, we show that this is especially true in contexts in which participants additionally have to choose between different options. We probed eye movement preparation by measuring saccade latencies to differently rewarded single targets (single-trial) appearing left or right from fixation. In choice-trials, both targets were displayed and participants were free to decide for one target to receive the corresponding reward. In blocks without choice-trials, single-trial latencies were not or only weakly affected by reward. With choice-trials present, the influence of reward increased with the proportion and difficulty of choices and decreased when a cue indicated that no choice will be necessary. Choices caused a delay in subsequent single-trial responses to the non-chosen option. Taken together, our results suggest that reward affects saccade preparation mainly when the outcome is uncertain and depends on the participants’ behavior, for instance when they have to choose between targets differing in reward.

## Introduction

Humans frequently decide where to look next. We shift our gaze 2–3 times a second by saccadic eye movements, each time choosing a different region or object of the visual scene for high acuity processing. This qualifies the oculomotor system as a suitable candidate to study decision-making in humans and other primates^1,2^. The selection of a particular target over others as well as the time required to initiate an eye movement (latency) are both informative about the underlying decision process.

Saccade latencies are not only influenced by low-level stimulus features^3,4^, but also by motivational factors like reward: Monkeys initiate saccades earlier and with higher peak-velocities when they expect a reward compared to non-rewarded saccades and reduced latencies are preceded by a modulated discharge rate of neurons in several brain areas^5–9^. To maximize outcome in decision-making, an option’s expected value (EV), the combination of reward magnitude and probability, has to be considered. Indeed, neural activity in the lateral intraparietal area (LIP) covaries with both reward magnitude and probability^10^. In humans, EV scales with activity of frontal areas^11–13^.

Despite this clear neurophysiological evidence that brain activity scales with reward, there are contradictory findings about its influence on eye movement preparation. Some studies did not find effects on saccade latencies in monkeys^10,14^ or reported changes in peak-velocity rather than latency when investing the effect of reward in humans^15,16^. However, there is also evidence favoring a modulation by reward. Two studies^17,18^ investigated whether saccades are influenced by a target’s EV. They showed that when two targets were presented (two-target trial), humans^17^ and monkeys^18^ more frequently chose the highly rewarded target. When one target was presented (single-target trial), latencies were affected by reward magnitude, but showed a stronger linear relationship with EV. This led to the conclusion that a representation of EV is incorporated in saccade preparation.

Where might these contradictory findings come from? A specific feature of the studies reporting an influence of EV on saccade preparation^17,18^ was the combined recording of several different trial types in the same experiment. Whereas latency analyses were based on responses to single-targets, additional trials were recorded in which participants had to choose among two targets (two-target trials) or trials with a distractor flashed before onset of the saccade target (distractor trials). These different trial types might have interacted: there is ample evidence that inter-trial priming can affect saccade metrics, especially when a competition between several targets is involved^19–21^.

Here, we investigated the hypothesis that effects of reward on saccade preparation are modulated or caused by interleaved choices between multiple targets. We measured saccade preparation by means of saccade latencies to single-targets (single-trials) and varied the proportion of interleaved choices (choice-trials) in a block. It is important to note that choice-trials were only included as independent variable: All results are based on latencies in single-trials. Differences in latencies to less and highly rewarded targets were present in blocks with interleaved choices – and mostly absent in blocks where participants never made a choice. The magnitude of this effect increased with increasing proportion and difficulty of choices. Choices caused a delay in subsequent saccade responses to the non-chosen target. Modelling latency distributions suggested that this delay was due to a reduced baseline level in the response signal.

## Results

### Latency differences between less and highly rewarded targets

**Figure 1:**
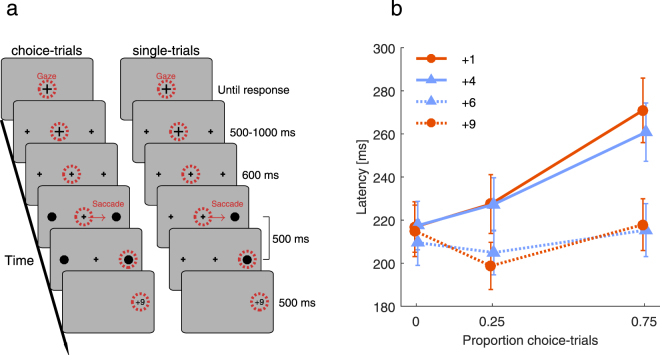
The effect of choice- on single-trials. (**a**) Trial procedure for choice- (left column) and single-trials (right column). Participants started trials by fixating the cross at the screen center (red dashed circle, not shown in the experiment) and simultaneously pressing a button on a keyboard. Two placeholders appeared in the periphery and after a random interval, the central fixation cross changed its size to indicate the onset of the target(s) in 600 ms. In single-trials, one target (black dot) replaced one of the two placeholder crosses and was displayed for 500 ms. Participants received a reward if they saccaded to the target during its presentation. In choice-trials, both placeholders turned into dots and participants could choose which target to saccade to in order to obtain the corresponding reward. Each side was either assigned a highly or a less rewarded target and participants were informed about the distribution of rewards before starting each block. (**b**) Latency in single-trials as a function of the proportion of choice-trials in the same block. Dashed lines refer to the highly rewarded, solid lines to the less rewarded targets. Blue colors and triangles denote the small, orange colors and circles the large reward difference. Error bars are 95% confidence intervals. Values are offset horizontally for better visibility.

In Experiment 1, we tested the hypothesis that saccade preparation in response to rewarded single-targets is modulated by the presence of choices which participants have to make in a block. To this end, we measured single-trial saccade latencies (Fig. 1a) in blocks without choice-trials (0%) or in blocks with different proportions of choice-trials randomly interleaved (25%, 75%). In single-trials, one target appeared at 15° eccentricity either left or right from fixation. Participants had to saccade to the target within 500 ms to receive the reward. In choice-trials, both targets were displayed and participants were free to decide for one target to obtain the corresponding reward. In every block, each hemifield was assigned either a highly or a less rewarded target. Across blocks, the difference in reward magnitude between the opposite hemifields could be either small (4 vs 6) or large (1 vs 9).

Saccade latencies from single-trials are shown in Fig. 1b. With an increasing proportion of choice-trials (0%, 25%, 75%) latency differences between less and highly rewarded targets increased for the large (2, 29 and 52 ms) and small reward difference (8, 22 and 45 ms), *F*(2,48) = 49, *p* < 0.001 (interaction proportion choice-trials × reward magnitude). This was mainly because latencies to less rewarded targets increased linearly with an increasing proportion of choice-trials, *F*(1,24) = 83.86, *p* < 0.001. Without choice-trials, latencies between less and highly rewarded targets did not differ significantly for the large reward difference: *t*(24) = 0.43, *p* = 0.671, but they did for the small if not Bonferroni-corrected: *t*(24) = 2.12, *p* = 0.045 (α’ = 0.05/6 [3 proportion choice-trials × 2 reward differences] = 0.0083). The corresponding Bayes factor (BF) favored the null hypothesis (i.e. reward does not influence latencies) for the large, *BF* = 0.23, but there was no conclusive evidence for the small reward difference, *BF* = 1.38. With 25% choice-trials, however, latency differences were significantly larger than without choice-trials, large: *t*(24) = 6.76, *p* < 0.001, small: *t*(24) = 4.02, *p* < 0.001. Compared to 25%, latency differences were even more pronounced with 75% choice-trials for the small reward difference, *t*(24) = 3.29, *p* = 0.003, but failed to reach significance for the large reward difference, *t*(24) = 2.29, *p* = 0.031, *BF* = 1.88. We found no evidence for an effect of reward difference (all *ps* > 0.4).

**Figure 2:**
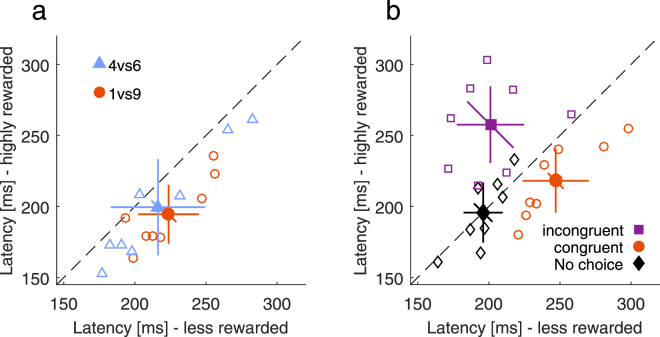
Latency delays are caused by choice-trials. (**a**) Results of Experiment 2. Single-trial latency for the highly versus less rewarded target when saccade frequency is equated for both hemifields. Open markers denote individual data and filled markers denote the mean with 95% confidence intervals. Diagonal error bars represent the error of the differences between high and low reward and have to be compared to the identity line. (**b**) Results of Experiment 3. Single-trial latency towards the highly compared to the less rewarded target when the reward in choice-trials was congruent (orange circle), incongruent (purple square) or without choice-trials (black diamond).

In Experiment 1, we mainly found differences in saccade latencies between less and highly rewarded targets when choices were interleaved. Because participants consistently chose highly rewarded targets, this observation could arise due to the choices themselves or because higher choice-trial proportions also implied lower saccade frequencies to the less rewarded (i.e. non-chosen) target. In Experiment 2, we eliminated this imbalance in saccade frequency by altering the frequency of single-trials to each target so that participants moved equally often to both targets if they always chose the highly rewarded target in choice-trials. Even with equated saccade frequency, participants still showed longer latencies to less rewarded single-targets, that is, non-chosen targets, *F*(1,7) = 123.97, *p* < 0.001 (Fig. 2a; main effect reward magnitude). Latency differences were 29 ms for the large, *t*(7) = 6.30, *p* < 0.001, and 17 ms for the small reward difference, *t*(7) = 4.27, *p* = 0.004, and thus similar to Experiment 1. Like in Experiment 1, we did not find evidence that reward differences affected latencies (all *ps* > 0.1).

We compared latency differences from Experiment 2 and the 25% choice-trial condition in Experiment 1. Experiments are identical with regard to choice-trial probability, but differ in saccade frequency. A 2 × 2 ANOVA with the factors reward difference (within) and experiment (between) revealed no significant main effect of experiment, *F*(1,31) = 0.39, *p* = 0.565, *BF* = 0.31. In a similar ANOVA, we compared latency differences from Experiment 2 with the 0% choice-trial condition in Experiment 1. Here, conditions from both experiments include the same saccade frequency, but differ with respect to choice-trial probability. Latency differences were larger in Experiment 2, *F*(1,31) = 24.61, *p* < 0.001, *BF* = 29.72. This suggests that latency differences between less and highly rewarded single-targets in blocks with interleaved choices occur even when overall saccade frequencies are matched for both targets.

To examine whether choices modulated the reward effects on saccade preparation or whether they caused them, we performed Experiment 3 where choice- and single-trial rewards were either incongruent or congruent, or where choice-trials were absent. In the congruent condition, highly rewarded targets for single- and choice-trials were presented in the same hemifield (equivalent to Experiment 1), whereas in the incongruent condition highly rewarded single- and choice-trials targets were presented in opposite hemifields. If the presence of choice-trials caused latency differences in single-trials, then single-trial latencies should only depend on which target is preferred in choice-trials and should be independent of the actual single-trial reward. Figure 2b shows mean and individual latencies for the different congruency conditions. Without choice-trials, latencies in both reward conditions perfectly coincided (196 ms) and did not differ significantly, *t*(7) = 0.06, *p* = 0.956, but the corresponding BF did not provide conclusive evidence, *BF* = 0.37. Instead, the effect of reward depended on the level of congruency, *F*(2,14) = 21.54, *p* < 0.001 (interaction reward magnitude × congruency). With congruent choice-trials present, latencies to less rewarded single-targets were increased by 29 ms (SD = 13 ms), *t*(7) = 6.2, *p* < 0.001. This pattern was reversed with incongruent choice-trials (M = −57 ms, SD = 39 ms), *t*(7) = 4.11, *p* = 0.005. Increased latencies in single-trials thus did not depend on single-trial reward itself, but on reward in choice-trials and therefore on which target was chosen. It thus seems that choices caused rather than modulated, the observed reward effects in single-trials.

### The non-chosen target is inhibited in the subsequent single-trial

**Figure 3:**
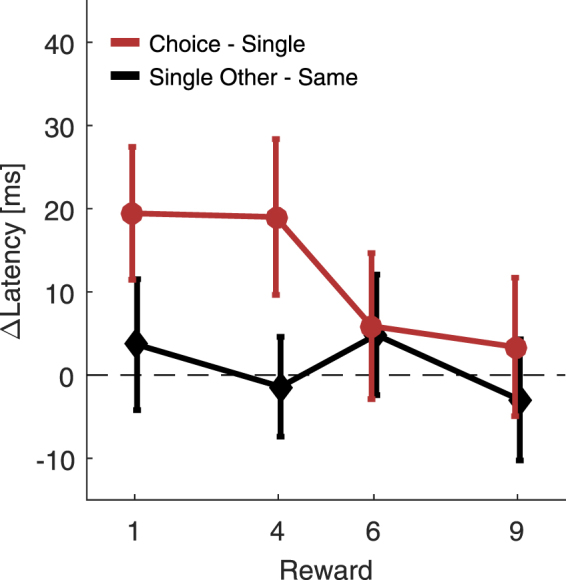
Inhibition of the non-chosen target. N-1 effects from Experiment 1. The difference in single-trial latency when the previous trial was a choice- compared to a single-trial (red circles). After a choice-trial, latencies to less rewarded single-targets, thus to the non-chosen target, are increased. This cannot be attributed to a change in saccade direction, because no such delay occurs when the previous trial was a single-trial in the opposite versus the same direction (black diamonds).

Confronted with a choice, one could either increase saccade preparation towards the highly rewarded and thus chosen target or one could inhibit the less rewarded and thus non-chosen target (or any combination of more mechanisms, see discussion). The former case predicts lower latencies when highly rewarded single-trials follow a choice-trial, whereas the latter case predicts increased latencies when less rewarded single-trials follow a choice-trial. The latter case appears more likely, given that we mainly found increased latencies for the non-chosen target. To test whether inter-trial effects contributed to our results, we reanalyzed the 25% choice-trial condition of Experiment 1 with regard to previous trial effects. We compared single-trials following a choice-trial with single-trials following a single-trial (Fig. 3). After choice-trials, saccades were initiated later when the upcoming trial was a single-trial to the non-chosen target, *F*(1,24) = 11.82, *p* = 0.002 (main effect reward magnitude). This cannot be attributed to a change in saccade direction, because there is no such difference when a previous single-trial was directed in the other or in the same direction, *F*(1,24) < 0.01, *p* = 0.954. An ANOVA which included both trial sequences, revealed an interaction of trial sequence with reward magnitude, *F*(1,24) = 7.13, *p* = 0.013 (i.e., an interaction of the black and red line in Fig. 3). Thus, in single-trials with less rewarded targets of either 1, *t*(24) = 4.77, *p* < 0.001, or 4 points, *t*(24) = 3.98, *p* < 0.001, saccades were significantly slower after a choice-trial. This suggests that the non-chosen target is inhibited in choice-trials, affecting the subsequent single-trial. We found no evidence that this effect increased with reward difference, *F*(1,24) = 4.01, *p* = 0.057.

### Adaptive inhibition of the non-chosen target

Is the delay of saccades to the non-chosen target an adaptive behavior? If yes, then it should scale with the necessity to inhibit the less rewarded target in choice-trials. One possibility to manipulate the necessity for inhibition would be to change the relative salience of both choice targets. When the less rewarded target has a higher contrast than the highly rewarded one, stronger inhibition is required to make an optimal saccade and thus obtain the high reward. Any location-based inhibition should then propagate to single-trials and lead to larger latency differences. The opposite pattern should be observed when the highly rewarded target is more salient. A second possibility to manipulate the required inhibition would be cueing the upcoming trial type. If participants know that the next trial will be a single-trial, they can refrain from maintaining inhibition and rely on a purely visually evoked saccade instead. This, however, would require that the inhibition could be modulated by top-down control. We tested these two possibilities in Experiment 4 and 5.

**Figure 4:**
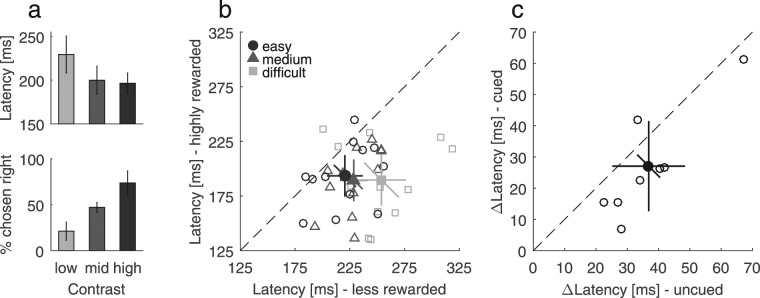
Adaptive inhibition of the non-chosen target. (**a**) Control latency task (top panel) and control choice task (lower panel) of Experiment 4, both without reward. With increasing target contrast, latency in single-trials decreased and the target with the higher contrast was preferably chosen in choice-trials. (**b**) Latency to the highly versus the less rewarded target for the different levels of difficulty (Experiment 4). The difference in single-trial latency increased with increasing difficulty in choice-trials. (**c)** Results of cueing (Experiment 5). Latency differences between less and highly rewarded single-trials were reduced when single-trials were cued compared to uncued.

In Experiment 4, we aimed to assess whether single-trial latency differences increase with the difficulty to saccade to highly rewarded targets in choice-trials. To this end, we changed the contrast of both choice-trial targets so that the contrast of the highly rewarded target was lower (difficult condition), higher (easy condition) or identical (medium condition). Beforehand, we measured two control conditions as a manipulation check (Fig. 4a). First, in the choice control task, participants had to choose one out of two targets which were either identical or different in contrast without receiving a reward. The probability of choosing targets on the right was lowest, when left targets had higher contrasts (M = 0.21, SD = 0.17), it was around chance when both contrasts were identical (M = 0.47, SD = 0.09) and highest when right targets had higher contrasts (M = 0.74, SD = 0.23), χ²(2) = 12.17, *p* = 0.002. Second, in the latency control task, we measured latencies to single-targets of different contrasts. Latencies decreased from 229 ms (low contrast) over 200 ms (medium contrast) to 196 ms (high contrast), *F*(2,22) = 15.91, *p* < 0.001. Compared to medium contrasts, latencies were increased for lower contrasts, *t*(11) = 4.7, *p* = 0.001, but they were not significantly decreased for high contrasts, *t*(11) = 0.81, *p* = 0.433.

Figure 4b shows individual and mean latencies for less and highly rewarded targets and for the three difficulty levels. Again, we found higher latencies to less rewarded targets, *F*(1,11) = 23.22, *p* = 0.001. Latency differences between less and highly rewarded targets were modulated by difficulty, *F*(2,22) = 7.24, *p* = 0.011. Compared to medium difficulty, latency differences were increased for the difficult condition, *t*(11) = 2.23, *p* = 0.047, and decreased for the easy condition, *t*(11) = 2.35, *p* = 0.038. Two separate ANOVAs suggested that difficulty affected latencies to less rewarded targets, *F*(2,22) = 8.39, *p* = 0.002, but not to highly rewarded targets, *F*(2,22) = 0.29, *p* = 0.751, *BF* = 0.2. Moreover, the probability to miss less rewarded single-trials increased with choice-trial difficulty, from 5% (easy), over 8.6% (medium) to 23.3% (difficult), *χ*²(2) = 15.2, *p* = 0.001. Misses were either due to too late (42.2%), too early (22.6%) or wrong saccades (35.2%). There was only one missed trial (<0.1%) in highly rewarded single-trials.

To test whether this behavior is not only adaptive with regard to low-level stimulus features, but also with regard to top-down processes, we cued half of the single-trials in Experiment 5. If there is a contribution from a top-down component, for example a preparation for an upcoming choice-trial, then differences in single-trial latencies between the chosen and non-chosen targets should be reduced by cueing. Figure 4c shows differences in saccade latencies, for cued compared to uncued single-trials. Latency differences were 37 ms without and 27 ms with cue. Wilcoxon signed-rank tests revealed that the latency difference was above 0 in both conditions, *Z* = −2.52, *p* = 0.012, but reduced by the presence of a cue, *Z* = −2.1, *p* = 0.036. This indicates that there is a voluntary component contributing to the observation of delayed saccades, yet it cannot fully account for it. In sum, the delay reduction by cueing (Experiment 5) and the delay increase with increasing difficulty (Experiment 4) point out that the inhibition of the non-chosen target is an adaptive behavior influenced by top-down and bottom-up factors.

### Decreased baseline level for the non-chosen target in single-trials

In order to identify likely neural mechanisms which can explain the delay of saccades to single-targets due to interleaved choices, we recorded the whole latency distribution for two participants (Experiment 6) and fitted the LATER model^22,23^ to the single-trial data. The LATER model is helpful in pointing out potential neural mechanisms of motor responses and decision making, on the basis of reaction time distributions. It assumes that for every response (here single-trial saccade) at stimulus onset, evidence is accumulated starting from a baseline level ${\theta }_{0}$ with an average rate of rise *µ* until a response threshold ${\theta }_{T}$ is reached. Within one trial the accumulation rate rises constantly but varies across trials with a Gaussian standard deviation *σ*. Several studies identified such evidence accumulation in the primate brain^24,25^ that can account for saccade latency distributions. With behavioral data however, it is only possible to obtain information about the threshold height, that is, the difference between baseline level and response threshold, *θ* = *θ*_*T*_ − *θ*_*0*_. Since there is physiological evidence that the baseline firing rate in saccade related areas represents economic decision variables as reward and target probability^10,25^ and saccades are initiated once the neural activity reaches a constant threshold^24^, we fixed the response threshold to an arbitrary value. The three remaining parameters are the baseline level, *θ*_*0*_, the accumulation rate, *µ*, and its variability, *σ*.

To find out which of these three parameters can most likely explain the latency differences between conditions, we abided by the following procedure: For every individual, we applied a bootstrap procedure with 100 iterations. For every iteration, we fitted three versions of the model in which we allowed one of the parameters to vary across conditions while the remaining two were kept identical across conditions. We then used information weights^26^, derived from the Bayesian information criterion (BIC) to compare the three model versions and thus to identify which parameter is best in explaining the latency differences across conditions. Information weights can range from 0 to 1 and higher values speak in favor of a particular model.

**Figure 5:**
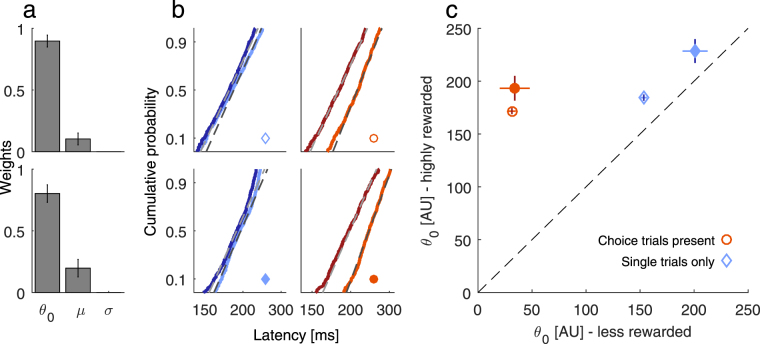
Results from the LATER model. (**a**) Information weights for the different model fits when each of the three parameters was allowed to differ across conditions. Values represent the mean weight with 95% confidence intervals across 100 Bootstrap samples. Each row (consistent with **b**) represents a different participant. For both participants, the ${\theta }_{0}$ parameter, baseline level, was best in describing differences across conditions. (**b**) Reciprobit plots of single-trial latency distributions to the highly (dark colors) and the less rewarded target (brighter colors) when choice-trials were present (right column; orange/red colors) or absent (left column; blue colors). Each row represents a different participant. Dashed lines are model fits for the high (bright gray) and low reward (dark gray). Dots indicate which marker is used to plot the ${\theta }_{0}$ parameters in (**c**). (**c**) Scatterplot of baseline levels, ${\theta }_{0}$, for the different conditions obtained by the model fit. Values represent the mean with 95% confidence intervals across 100 Bootstrap samples. Participants can be told apart by open or filled symbols (same as in **b**). With choice-trials present (orange circles), baseline levels for less rewarded single-trial targets were strongly reduced.

With regard to average latencies, we replicated our main findings also with the more extensive measurements with these two participants: Without interleaved choices, average single-trial latencies were M = 192 ms for the less and M = 187 ms for the highly rewarded target. With choice-trials present, latencies were M = 192 ms for the highly and M = 228 ms for the less rewarded target. For both participants, information weights (Fig. 5a) were highest for the ${\theta }_{o}$ parameter (baseline level). Thus, changes in ${\theta }_{o}$ were best in explaining differences in latency distributions between conditions. Cumulative probability plots of latency distributions together with model fits are plotted in Fig. 5b. Without choice-trials, baseline levels for less rewarded single-trials were reduced by 12 and 17% relative to baseline levels for highly-rewarded targets. With choice-trials present, baseline levels were reduced by 82% (Fig. 5c) for both participants. Technically, this suggests that either a lower baseline level, an increased response threshold or both are most likely to explain delayed saccades to non-chosen targets.

## Discussion

In this study we investigated whether saccade preparation to single-targets is influenced by interleaved choices among two targets differing in reward and if this is able to account for differential previous results on the modulation of saccade latencies by reward. In blocks without choices (Experiment 1 and 3), we only found a comparatively small effect of reward on saccade latencies that was only significant in one (only without correction for multiple testing) out of three cases. When choices were present, reactions to less rewarded single-targets were delayed and the magnitude of this delay increased significantly with the proportion of choice-trials, both for saccades (Fig. 1b) and button presses^27^. When changing the reward congruency between choice- and single-trials, latency differences in single-trials depended on the reward assignments in choice- rather than in single-trials (Fig. 2b). Moreover, latency differences were adaptive because they scaled with the necessity to inhibit saccades which do not maximize reward during choices (Fig. 4b) and decreased when upcoming single-trials were cued in advance (Fig. 4c), suggesting the contribution of both, bottom-up and top-down factors. Increased latencies to less rewarded single-targets can be explained in terms of a reduced baseline level (Fig. 5). Although a difference in response threshold could technically also account for the observed latency difference, this is unlikely given that saccades are executed at a constant threshold^24^.

Taken together, our results suggest that information about reward might not always be incorporated for the preparedness of motor responses like saccadic eye movements. This does neither suggest that it is not represented in the brain, nor that it does not affect behavior. Rather, it suggests that reward affects preparation of saccades mostly when it is behaviorally relevant as in choice-trials and less so when it is behaviorally irrelevant as in single-trials. When responding to single-targets without strong temporal urgency, there is no necessity to optimize behavior, for instance, by preferring one target location over the other. Thus, the modulation of latencies in single-trials appears to be a direct effect of target selection and mostly no (or only an indirect) effect of reward per se.

Many studies have shown that reward influences oculomotor behavior. Monetary and non-monetary reward alters eye movement behavior, by changing saccade latencies^5,28–30^, kinematics^15,31,32^ and target selection^33–35^. Most of these studies however have compared rewarded to unrewarded behavior and did not include different levels of reward. When rewards of different magnitudes can be obtained, saccade endpoints are closer to high than to low reward targets^36^ and maximize gain^33^, the microsaccade rate scales with value^37^ and saccade vigor decreases with advanced discounting of rewards^16,38^. Here, looking at saccade latencies without interleaved choices (Experiment 1 & 3), we found no significant evidence for a direct influence of value in two out of three conditions. Bayesian analyses provided evidence for the notion that reward magnitude does not affect latencies in one out of three cases and inconclusive evidence in the remaining two. In Experiment 6, reward influenced baseline levels even without choice-trials. This might point out that latency distributions are more sensitive to reward than average latencies. However, congruent with the average latency differences in the other experiments, modulations of baseline levels were much larger with choice-trials. Thus, in total this suggests that reward alone influences latencies only weakly or not at all. Moreover, the magnitude of reward differences did not modulate latencies (Experiment 1 & 2). This, together with the observation that response delays to less rewarded single-targets can be varied by the amount of inhibition required to perform a reward-maximizing choice (Experiment 4), suggests that participants tried to make an optimal choice, no matter how big the gain or loss.

A previous study^17^ reported a linear relation of saccade latency and EV. However, because choice- and single-trials were mixed in this study, it is unclear whether this link would persist in the absence of choices. The here reported inter-trial dependency might also have affected oculomotor and neural findings in monkeys^18,39^. A recent study tested whether microsaccade behavior also varies as a function of EV^37^. The authors reanalyzed their previously collected monkey data mixing choice and single responses^18^ and recorded new human data for single-trials only. Both, humans’ and monkeys’ microsaccades were biased by the subjective target value. This points out that microsaccades seem to represent value irrespective of whether choices are interleaved or not. Unfortunately, the authors did not report saccade latencies for the human data. This could have been an indication whether or not EV can affect saccade preparation in the absence of interleaved choices.

Our results suggest that target selection modifies subsequent saccade preparation. There is converging evidence that attentional control is not only influenced by stimulus properties (bottom-up) or current goals (top-down), but also by a bias to attend previously selected items^40,41^. For example, inter-trial priming effects seem to require attentional selection^42^ and can either be facilitating or inhibitory. Facilitating effects can be observed in visual search when the search target, distractors or their particular features are identical to the preceding trial, leading to shorter reaction times^43–45^. Inhibitory effects occur in conjunction with distractors, for example, saccades curve away from previous distractor locations^19^ or in the negative priming paradigm, when the identities of target and distractor are exchanged between trials^46^. The present study extends findings on inter-trial priming by showing that a selection between two differentially rewarded targets does not facilitate a subsequent response to the chosen one but inhibits a response to the non-chosen one.

We interpret our results in terms of an inhibition of the less rewarded target. Theoretically, the fact that latencies to less rewarded targets increased with an increasing proportion of choices in Experiment 1 does not necessarily imply that these targets are selectively inhibited. Other combinations of several inhibitory and facilitating mechanisms could also explain this pattern: the presence of choices might generally slow down latencies and, simultaneously, selectively speed up responses to highly rewarded targets. In this case, these two mechanisms might cancel each other out for highly rewarded targets, whereas delays towards the less rewarded one would become observable. However, this alternative interpretation seems unlikely because of two other findings: first, the analysis of inter-trial effects showed that responses to less rewarded targets were slowed down after choice-trials, but responses to highly rewarded targets remained unaffected. However, a potential facilitation for highly rewarded targets should have been observable here. Second, the same argument is true for the findings of Experiment 4, where choice-trial difficulty selectively modulated latencies to less but not to highly rewarded targets (Fig. 4b). These two findings favor a selective inhibition of less rewarded targets, but we cannot rule out that other mechanisms are also involved.

Differences between conditions with different proportions of choice-trials (Experiment 1) could theoretically be explained by changes in saccade frequency. Although we cannot dismiss this interpretation, we consider it unlikely, given that latency delays with 25% choice-trials had the same magnitude when we equated saccade frequencies in both directions (Experiment 2). Moreover, studies showing influences of probability on saccade latencies^23,47^ employ hundreds of trials for every probability condition and dismiss the first 100 trials or more, whereas our blocks in Experiment 1 consisted of only 80 trials.

In Experiment 5 we tested whether our results are influenced by the expectation of an upcoming choice-trial. We cued single-trials to eliminate the expectation of an upcoming choice-trial. If expectation could fully explain our data, latency imbalances should have completely disappeared when upcoming single-trials were cued. However, latency differences were only reduced but not eliminated, suggesting that expectation can only partially explain our findings. Because we only manipulated expectation on a short-term timescale (trial-wise), we cannot exclude the possibility that expectation operating on longer timescales (block-wise) influenced our data but was unaffected by cueing. Nonetheless, our findings cannot be explained by (long-term) expectation alone, given that we found strong inter-trial effects within the same block. However, expectation (short-term and long-term) will have likely added up with inter-trial effects and resulted in delayed saccades to the less rewarded target.

In conclusion, our findings suggest that there is no or only a weak direct connection between reward and saccade preparation to single-targets. A decision between two reward-associated targets leads to a subsequent delay in responses to the non-chosen option. The amount of delay depends on the difficulty to make an optimal, that is, reward-maximizing decision in choice-trials. We propose that these changes in saccade preparation occur due to the subsequent inhibition of the non-chosen target and the expectation of an upcoming choice-trial. This is reflected by a reduced baseline level in the response signal. These results suggest that reward affects saccade preparation particularly if it is behaviorally relevant, for instance if a choice has to be made.

## Methods

### Participants and apparatus

In total, 47 students from Marburg University aged 19–29 years (M = 23 years) participated in this study (30 female, 17 male). All of them had normal or corrected to normal vision and gave prior informed consent. Participants were paid for participation (8€/h) and received additional reward based on their performance. All experiments were conducted in accordance with the ethical standards laid down in the 1964 Declaration of Helsinki and were approved by the local ethics committee LEK FB06 at Giessen university (proposal number 2013–0020). We recorded 25 participants for Experiment 1, 8 participants for Experiment 2, 3 and 5, 12 participants for Experiment 4 and 2 for Experiment 6. Experiments were conducted using the Psychtoolbox^48^ in MATLAB (The Mathworks, Natick, MA, USA) and presented on a VIEWPixx monitor (VPixx Technologies Inc., Saint-Bruno, Quebec, Canada) at a viewing distance of 60 cm. The monitor had a spatial resolution of 1920 × 1080 pixel and a size of 51.5 × 29 cm. We recorded eye movements of the right eye using a desktop mounted EyeLink 1000 (SR Research Ltd., Ontario, Canada) with a sampling rate of 1000 Hz and the Eyelink Toolbox^49^.

### General methods

At the beginning of each trial, a black fixation cross with a diameter of 0.5° appeared at screen center on a gray background (Fig. 1). Participants could start trials by pushing the space bar on a keyboard while maintaining fixation. Two crosses (placeholders) with a diameter of 0.25° appeared both left and right from fixation at an eccentricity of 15°. After a random interval (500–1000 ms), the central fixation cross changed its size to 0.25° indicating the onset of the target after additional 600 ms. Targets were dots with a radius of 0.25° and were presented for 500 ms. In single-trials, one dot replaced one of the placeholders, whereas in choice-trials both placeholders were replaced by dots. Participants were instructed to maintain fixation until target appearance and then saccade to a target while it was presented. If they succeeded, their reward for that trial was shown at the target location after target offset. If participants did not make saccades or made saccades to placeholders, they received no reward. Rewards were score points (1, 4, 6 or 9) which were converted into monetary reward at the end of the experiment (1€ for 500 points). At the beginning of each block, participants were informed about the distribution of reward to each hemifield and the relative probability of choice and single-trials. For every experiment, the order of blocks was balanced across participants.

### Experiment 1

Experiment 1 tested the hypothesis that the effect of reward on saccade latencies in single-trials is modulated by the presence of interleaved choice-trials. We varied the proportion of choice-trials within one block (0%, 25%, 75%). In every block, a fixed reward was assigned to each target/hemifield and rewards were identical for choice- and single-trials. Rewards summed up to 10 score points with one target receiving a higher reward (6 or 9, ‘highly rewarded target’) than the other (4 or 1, ‘less rewarded target’). The reward difference between the two hemifields could be large (1 vs 9) or small (4 vs 6). The experiment thus comprised the three factors (i) choice-trial probability (0%, 25%, 75%), (ii) reward magnitude (highly or less rewarded) and (iii) reward difference (large or small). The trial order was randomized and single-trials to both hemifields appeared equally often. Every combination of choice-trial probability and reward difference was recorded in a block of 80 trials. In total, every participant completed 480 trials and could receive up to 5.60€ reward. The experiment lasted 60–90 minutes.

### Experiment 2

To show that latency differences caused by choice-trials cannot be explained by a higher saccade probability to highly rewarded targets, we increased the single-trial probability to the less rewarded side. Every participant completed two blocks, one for a small (4 vs 6) and one for a large (1 vs 9) reward difference. Blocks consisted of 120 trials and contained 30 choice-trials (25%). The remaining 90 trials were single-trials, 30 to the highly rewarded and 60 to the less rewarded side. Consequently, participants would saccade equally often to both hemifields if they always chose the highly rewarded target in choice-trials. If they did not, the saccade probability to the less rewarded target would be even higher than 50%.

### Experiment 3

To test whether the presence of choice-trials modulates or causes the effects of reward on saccade preparation, we changed the reward correspondence between choice- and single-trials. Every participant completed three blocks of 120 trials, all with a high reward difference (1 vs 9). In one block, the highly rewarded side for choice- and single-trials was identical (congruent condition), like in Experiment 1. In another block, the highly rewarded side for choice-trials was the less rewarded side in single-trials (incongruent condition). Both, the congruent and incongruent condition contained 75% of choice-trials. In a third block there were only single-trials.

### Experiment 4

In order to assess whether the latency modulation due to choices is adaptive, we varied the choice difficulty by changing the contrast of both targets. All targets were darker than the background and Michelson contrasts were 0.5 (black), 0.2 and 0.08. In the difficult condition, the contrast of the highly rewarded target was 0.08 while the other had a contrast of 0.5. It was the other way round for the easy condition. In the medium condition, both targets had identical contrasts (0.2). The same contrast of 0.2 applied to all fixation crosses, placeholders and targets in single-trials. To make the transition from placeholder to target less salient for the low contrast condition, placeholders remained visible on top of the target during the whole trial for all conditions. Every condition comprised 120 trials. As a manipulation check, we additionally recorded a choice control task and a latency control task. The choice control task consisted of 60 choice-trials without reward but with either the same (0.2) or a different contrast (0.08 vs 0.5). The latency control task consisted of 120 unrewarded single-trials of the three different contrast levels.

### Experiment 5

To determine whether the effects observed in the previous experiments are caused by the expectation of an upcoming choice-trial, we cued half of the single-trials. The cue was a “1” displayed 1.3° above the central fixation cross. It appeared together with the peripheral placeholders and vanished after 200 ms. The whole experiment consisted of 280 trials, with 50% choice-trials and 25% of cued and uncued single-trials each.

### Experiment 6

To determine likely neural mechanisms for the interaction of choice- and single-trials, we measured latency distributions to single-trials with (50%) and without choice-trials interleaved and fitted the LATER model^22,23^ to the data. Blocks consisted of 100 trials and participants completed 10 blocks without and 20 blocks with choice-trials (3000 trials in total).

### Data and statistical analysis

We used the EyeLink 1000 algorithm to determine saccade onsets. Latencies were defined as the first saccadic sample with respect to target onset and successful target choice was defined as the first sample where the gaze was within a square region of 2° around the target. Trials with saccades initiated earlier than 100 ms or later than 450 ms after target onset were not considered for the final analysis of latencies. Across all experiments (apart from Experiment 4 where missing the target was a dependent variable), this happened in 1.59% of trials. Due to technical issues, some eye movement traces could not be saved in 2.99% of trials. These recording errors were evenly distributed across all experiments and conditions.

Normality of the data was assessed by Kolmogorov-Smirnov tests and by visually inspecting Q-Q-plots. Statistical tests on saccade latencies in Experiment 1–4 were done using repeated-measures ANOVA and post-hoc t-tests with Bonferroni-corrected α level. If sphericity was violated, we report corrected p-values according to Greenhouse-Geisser. We supplemented our analyses with Bayes factors^50^ (BF) when non-significant results were crucially relevant for interpreting the data. BFs were computed in R (3.3.2; R Development Core Team, 2016) using the BayesFactor package with default priors. BFs smaller one favor the null hypothesis and values greater one favor the alternative hypothesis. Evidence is stronger, the further BFs deviate from 1, with values between 0.33 and 3 being considered inconclusive evidence^51^. In Experiment 5, we compared latency differences using Wilcoxon signed-rank tests, because the data were not normally distributed. Performance values in Experiment 4 were compared using the non-parametrical Friedman test. Analyses were carried out in MATLAB, R and SPSS (Version 22, IBM Corp., Armonk, NY).

### Choice-trial behavior

In all experiments, we varied the presence of choice-trials as independent variable without being interested in the participants’ behavior in these trials. In choice-trials, participants almost always chose the target with the higher reward (e.g. in Experiment 1: M = 95.3%, SD = 2.5%; Experiment 3: M = 95%, SD = 3.1%) with similar latencies as in single-trials without choice-trials (Experiment 1: M = 214 ms, SD = 22 ms) or slightly elevated (Experiment 3: M = 224, SD = 22 ms).

### Data Availability

Data are publicly available at the doi:10.5281/zenodo.343881.
